# Extracorporeal cardiopulmonary resuscitation for adult out-of-hospital cardiac arrest patients: time-dependent propensity score-sequential matching analysis from a nationwide population-based registry

**DOI:** 10.1186/s13054-023-04384-y

**Published:** 2023-03-06

**Authors:** Yeongho Choi, Jeong Ho Park, Joo Jeong, Yu Jin Kim, Kyoung Jun Song, Sang Do Shin

**Affiliations:** 1 Department of Emergency Medicine, Seoul National University College of Medicine and Seoul National University Bundang Hospital, Seoul, Republic of Korea; 2 Disaster Medicine Research Center, Seoul National University Medical Research Center, Seoul, Republic of Korea; 3 Laboratory of Emergency Medical Services, Seoul National University Hospital Biomedical Research Institute, Seoul, Republic of Korea; 4 Department of Emergency Medicine, Seoul National University College of Medicine and Seoul National University Hospital, Seoul, Republic of Korea; 5 Department of Emergency Medicine, Seoul National University College of Medicine and Seoul National University Boramae Medical Center, Seoul, Republic of Korea

**Keywords:** Out-of-hospital cardiac arrest, Outcome, Extracorporeal membrane oxygenation, Propensity score

## Abstract

**Background:**

There is inconclusive evidence regarding the effectiveness of extracorporeal cardiopulmonary resuscitation (ECPR) for out-of-hospital cardiac arrest (OHCA) patients. We aimed to evaluate the association between ECPR and neurologic recovery in OHCA patients using time-dependent propensity score matching analysis.

**Methods:**

Using a nationwide OHCA registry, adult medical OHCA patients who underwent CPR at the emergency department between 2013 and 2020 were included. The primary outcome was a good neurological recovery at discharge. Time-dependent propensity score matching was used to match patients who received ECPR to those at risk for ECPR within the same time interval. Risk ratios (RRs) and 95% confidence intervals (CIs) were estimated, and stratified analysis by the timing of ECPR was also performed.

**Results:**

Among 118,391 eligible patients, 484 received ECPR. After 1:4 time-dependent propensity score matching, 458 patients in the ECPR group and 1832 patients in the no ECPR group were included in the matched cohort. In the matched cohort, ECPR was not associated with good neurological recovery (10.3% in ECPR and 6.9% in no ECPR; RR [95% CI] 1.28 [0.85–1.93]). In the stratified analyses according to the timing of matching, ECPR with a pump-on within 45 min after emergency department arrival was associated with favourable neurological outcomes (RR [95% CI] 2.51 [1.33–4.75] in 1–30 min, 1.81 [1.11–2.93] in 31–45 min, 1.07 (0.56–2.04) in 46–60 min, and 0.45 (0.11–1.91) in over 60 min).

**Conclusions:**

ECPR itself was not associated with good neurological recovery, but early ECPR was positively associated with good neurological recovery. Research on how to perform ECPR at an early stage and clinical trials to evaluate the effect of ECPR is warranted.

## Background

Extracorporeal cardiopulmonary resuscitation (ECPR) using venoarterial extracorporeal membrane oxygenation (VA-ECMO) has been introduced to treat patients with refractory out-of-hospital cardiac arrest (OHCA) of a cardiac origin [1]. However, whether ECPR is effective in the real world is unclear [2]. Meta-analyses and systematic reviews with pooled results have shown inconclusive results regarding the benefits of ECPR over conventional resuscitation in refractory OHCA [3–5]. Contradictory results have also been obtained in recent randomised studies [6, 7]. Trials investigating ECMO-facilitated resuscitation with immediate angiography for patients with refractory ventricular fibrillation were terminated prematurely due to its superiority over other interventions [7]. Trials investigating intra-arrest transport to a cardiac centre for ECPR and immediate invasive assessment for patients without return of spontaneous circulation (ROSC) were also terminated prematurely due to a lack of superiority in the primary endpoint [6].

Although current evidence for ECPR for cardiac arrest is mainly based on observational studies, most observational studies had design limitations and various biases [8]. It is important to make efforts to reduce the biases of observational studies on the effect of ECPR before confirmatory clinical trials. Propensity score matching analysis is one popular method to reduce bias in observational studies [9, 10]. Recent systematic review and meta-analysis of 6 propensity score-matched studies for ECPR was also inconclusive in that the better 30-day favourable neurologic outcome of ECPR was revealed in the pooled analysis, but not in the separate analysis for in-hospital cardiac arrest and OHCA [8].

For time-sensitive diseases such as OHCA, resuscitation time bias could be one of the most important to affect the validity of the analysis [11]. Since specific interventions such as ECPR could not be given after ROSC, such interventions are more likely to occur in non-resuscitated patients. Moreover, as the prolonged duration of resuscitation is related to worse outcomes [12], the intervention group is expected to show poorer outcomes than the non-intervention group. Conventional propensity score-matched analysis could not overcome this bias [13]. In addition, adjusting the CPR duration using traditional methods is likely to introduce biased results, and the direction of this bias can be difficult to predict [14]. Time-dependent propensity score matching is a method for risk-set matching to adjust for potential confounders and address the resuscitation time bias [12, 13, 15, 16]. If we use time-dependent propensity matching analysis, we could conduct a retrospective analysis with reducing resuscitation time bias. To the best of our knowledge, no existing study has evaluated ECPR using time-dependent propensity score matching analysis to date.

In this study, we aimed to investigate the association between ECPR and neurologic outcomes in OHCA patients using time-dependent propensity score matching analysis. Because the timing of ECPR has also been reported to be associated with survival outcomes [17], we also evaluated the association between ECPR timing and survival outcomes.

## Methods

### Study design and setting

This cross-sectional study was conducted using a prospectively collected nationwide emergency medical service (EMS)-based OHCA registry from Korea. This study was approved by the Institutional Review Board (IRB) of the study institution (IRB No. 1103-153-357). The requirement for informed consent was waived.

The National Fire Agency operates a prehospital EMS system exclusively in Korea. In cases of OHCA, all EMS providers can provide basic life support, and qualified EMS providers can provide advanced life support, including advanced airway management, intravenous catheter insertion, or epinephrine use under direct medical control. EMS providers have no authority to declare death or stop CPR unless there is ROSC, and all OHCA patients should be transported to the hospital. There is no prehospital ECMO programme in Korea; therefore, all ECPR procedures can be performed in a hospital.

The Korean Ministry of Health has designated the following three levels of emergency department (ED): level 1 (*n* = 36) and level 2 (*n* = 119), which provide the highest level of emergency care services with emergency physicians on staff all times, and level 3 (*n* = 261), which may be staffed by general physicians. All EDs generally perform acute cardiac care and post-resuscitation care in accordance with national guidelines national guidelines that were adapted from the American Heart Association (AHA) Guidelines for Cardiopulmonary Resuscitation and Emergency Cardiovascular Care [18, 19]. No major differences in post-resuscitation care recommendations were observed between the two guidelines. In 2020, 74 EDs conducted at least one extracorporeal life support (ECLS) intervention for OHCA patients (median [interquartile range] volume of ECLS for OHCA 4 (2–6). The decision to perform ECPR is determined by attending physicians at each centre, and eligibility criteria using age, comorbidities, and cardiac rhythm can be used according to the centre [20–22]. In most centres, ECPR is performed by thoracic surgeons or cardiologists rather than an emergency physician.

### Data source

The Korean OHCA Registry, which includes all EMS-assessed OHCA cases, was retrieved from the following four sources: the EMS run sheets for basic ambulance operation information, the EMS cardiac arrest registry, the dispatcher CPR registry for the Utstein factors, and the hospital medical record review registry for hospital care and outcomes. Medical record reviewers from the Korea Disease Control and Prevention Agency extracted recorded information on hospital care and outcomes from approximately 700 hospitals. Professional medical record review experts were trained to use the Utstein guidelines to conduct a medical record review of variables related to the aetiology, risks, and outcomes of OHCA. To ensure the quality of the medical record review process, a quality management committee of emergency physicians, epidemiologists, statisticians, and medical record review experts analysed the data every month while providing feedback to each medical record reviewer [23, 24].

### Study population

All EMS-treated OHCAs due to a medical cause in patients aged 18 years or older and received ECPR from January 2013 to December 2020 were included. Patients with OHCA due to non-medical causes such as trauma, poisoning, hanging, hypothermia or drowning were excluded from the study. Patients who achieved ROSC upon arrival at the ED, death on arrival (DOA) at the ED, or those who were not resuscitated (DNR) were excluded. Patients with missing information on ECPR, ECMO pump-on time, propensity score-related variables, and survival outcomes were also excluded.

### Outcomes

The primary endpoint of this study was survival with good neurological recovery, defined as a cerebral performance category score of 1 or 2 [25]. The cerebral performance category score was measured by reviewing the participants’ medical records at the hospital discharge points [26]. The secondary endpoint was survival until discharge.

### Variables and measurements

ECPR was the primary exposure and was defined as successful venoarterial ECMO implantation and a pump-on during the cardiac massage; therefore, ECMO pump-on time was documented as before the last ROSC.

We collected information on age, sex, medical history (diabetes mellitus, hypertension, heart disease, and stroke), place of cardiac arrest (public or others), and bystander CPR (yes or no). We also collected information on the type of initial cardiac rhythm (shockable or pulseless electrical rhythm, asystole), prehospital management (defibrillation, fluid administration, mechanical CPR, and advanced airway management [endotracheal intubation or supraglottic airway management] by EMS providers), response time interval (call to the arrival of the ambulance at the scene), scene time interval (arrival to departure from the scene), transport time interval (departure from the scene to arrival at the ED), any prehospital ROSC prior to ED arrival, percutaneous coronary intervention, and targeted temperature management. For targeted temperature management, only the data from the cases where an explicit body temperature control method and target body temperature were specified with core body temperature monitoring, were collected. ECPR-related variables, including the location of ECPR (ED, catheterisation laboratory, or others) and total ECLS duration (time from ECMO pump-on to ECMO turn-off time), were also collected.

### Statistical analysis

Categorical variables were compared using the χ^2^ test or Fisher’s exact test, and continuous variables were compared using the *t*-test or Wilcoxon rank-sum test, as appropriate.

We used a time-dependent propensity score and risk-set matching analysis to assess the association between ECPR and survival outcomes [13, 27, 28]. We calculated the propensity score based on a Cox proportional hazards model. In the Cox regression model, the time to ECMO pump-on time was the dependent variable, and we considered patients as censored when CPR was stopped for patients who did not receive ECPR. Time 0 was defined as the arrival of the patient to the hospital because the patients were at risk of receiving ECPR after this time point. The covariates included in the propensity score-predicting model were age (continuous), sex, past medical history (diabetes mellitus, heart disease, stroke), region, call time to EMS (year, weekday, hour), witnessed status, public place, bystander CPR, bystander defibrillation, insurance, region category, EMS time variable (response time interval, scene time interval, total time interval), initial rhythm measured by EMS, intravenous route, EMS epinephrine injection, mechanical CPR by EMS, advanced airway, defibrillation by EMS, and any prehospital ROSC prior to ED arrival.

Based on the predicted time-dependent propensity scores, sequential optimal 1:4 matching without replacement was performed using the *optmatch* package [13, 16, 29]. Patients who started ECMO at any given time interval after time 0 were sequentially matched with patients who were ‘at risk’ of receiving ECPR within the same minute. Since the number of patients at both extremes of the time to ECMO pump-on time was small, cases with 0–10 min, 11–13 min, 75–120 min, and 121 min or more were combined into group in this matching, resulting in a total of 70 time intervals (min 0–10, 11–13, each minute of 14–70, 75–120 with intervals of 5 min, longer than 120 min). In the entire cohort and time-dependent propensity score-matched cohort, we determined the standardised mean differences for each covariate to assess the balance of covariates between the groups according to ECPR. We considered a standardised mean difference of less than 0.25, which is a well-matched balance between matched cohorts after propensity score matching [30, 31].

After matching, a generalised estimating equation (GEE) model with a log link function was constructed to calculate risk ratios (RRs) with 95% confidence intervals (CIs) for ECPR and each outcome [27, 32]. GEEs were used to address potential within-pair correlation risk-set matching. As the effect of ECPR can vary according to the timing of ECPR [33], we additionally performed analyses stratified by the timing of ECPR (divided into 4 groups including 1–30 min, 31–45 min, 46–60 min, and 61–min groups). In the stratified analyses, the RRs and 95% CIs were estimated to determine the outcomes in the matched cohorts.

The study’s main analysis were stratified based on the initial cardiac rhythm because initial cardiac rhythm could differently affect the effectiveness of ECPR. For stratified analysis, we selected patients with the corresponding characteristics from the matched cohort and then picked the already matched pairs of ECPR and no EPCR groups. Therefore, sequentially matched pairs in each time stratum were not collapsed after the stratification [28]. Sensitivity analysis including additional adjustment for EMS arrival at scene to ED arrival time was also performed because those prehospital times which reflects low flow time could significantly affect survival outcomes. Statistical significance was set at P < 0.05, and all analyses were performed using R version 4.0.4 (www.r-project.org).

## Results

### Baseline characteristics

**Fig. 1:**
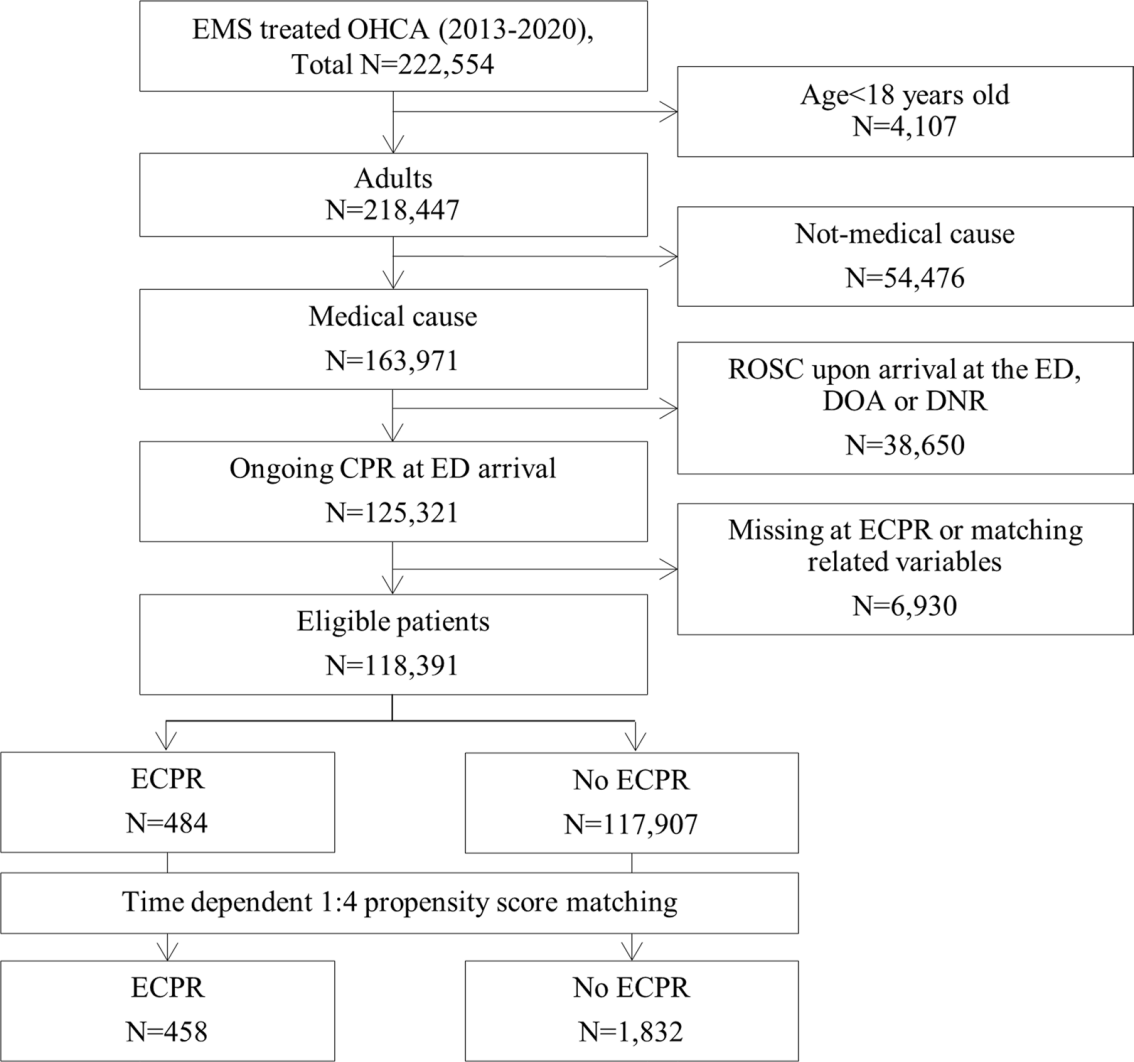
Patient flow. *EMS* Emergency medical service; *OHCA* Out-of-hospital cardiac arrest; *ROSC* Return of spontaneous circulation; *DOA* Death on arrival; *DNR* Do not resuscitate; *ECPR* Extracorporeal cardiopulmonary resuscitation

Among the 222,554 EMS-treated OHCA patients enrolled from January 2013 to December 2020, 104,163 were excluded because they were under 18 years of age, with non-medical causes, patients who achieved ROSC upon arrival at the ED, DOA, DNR, or missing ECPR-related variables or other matching variables. After exclusion, 118,391 patients with OHCA were included in the final analysis (Fig. 1). Among them, 484 (0.04%) underwent ECPR. Of the 118,391 patients, 2290 (458 in the ECPR group and 1832 in the no ECPR group) were matched after time-dependent propensity score matching.

The baseline characteristics of the original cohort according to ECPR and those of the matched cohort are presented in Table 1. Within the matched cohort, 252 (55.0%) and 1011 (55.2%) of patients were witnessed by laypersons, and 84 (18.3%) and 296 (16.2%) patients were witnessed by EMS in the ECPR group and no ECPR groups, respectively. The arrest occurred in a public place in 180 (39.3%) of the ECPR group and 686 (37.4%) of the no ECPR group. Bystander CPR was performed for 248 (54.1%) of the ECPR group and 1025 (55.9%) of the no ECPR group. Initial cardiac rhythm was shockable in 271 (59.2%) and 1052 (57.4%) of the ECPR and no ECPR groups, respectively. Any ROSC before to ED arrival was 52 (11.4%) in the ECPR group and 237 (12.9%) in the no ECPR group. Both groups were well-balanced in all the included variables (Table 1).

**Table 1:** Patient characteristics of the entire cohort and matched cohort

	Entire cohort			Matched cohort		
	No ECPR	ECPR	SMD	No ECPR	ECPR	SMD
	*N* = 117,907	*N* = 484		*N* = 1832	*N* = 458	
*Demographics*						
Age, mean (SD) [years]	70.1 (15.0)	55.2 (14.2)	1.022	56.4 (16.1)	55.5 (14.1)	0.059
Female sex	43,296 (36.7)	88 (18.2)	0.425	358 (19.5)	84 (18.3)	0.031
Urbanisation level of residence			0.586			0.067
Metropolitan	70,077 (59.4)	407 (84.1)		1538 (84.0)	382 (83.4)	
Urban	34,241 (29.0)	65 (13.4)		229 (12.5)	64 (14.0)	
Rural	13,589 (11.5)	12 (2.5)		65 (3.5)	12 (2.6)	
Year of arrest			0.277			0.107
2013–2014	23,093 (19.6)	90 (18.6)		292 (15.9)	87 (19.0)	
2015–2016	27,763 (23.5)	72 (14.9)		266 (14.5)	71 (15.5)	
2017–2018	31,104 (26.4)	139 (28.7)		575 (31.4)	132 (28.8)	
2019–2020	35,947 (30.5)	183 (37.8)		699 (38.2)	168 (36.7)	
*Past medical history*						
Diabetes	28,154 (23.9)	112 (23.1)	0.017	405 (22.1)	108 (23.6)	0.035
Hypertension	41,693 (35.4)	178 (36.8)	0.029	708 (38.6)	170 (37.1)	0.032
Heart disease	20,402 (17.3)	95 (19.6)	0.060	328 (17.9)	90 (19.7)	0.045
Stroke	10,789 (9.2)	18 (3.7)	0.223	69 (3.8)	18 (3.9)	0.009
*Circumstance of arrest*						
Day of arrest, weekend	34,500 (29.3)	103 (21.3)	0.184	342 (18.7)	100 (21.8)	0.079
Time of arrest, night	76,565 (64.9)	277 (57.2)	0.159	1035 (56.5)	262 (57.2)	0.014
Witness status			0.499			0.065
No	58,537 (49.6)	128 (26.4)		525 (28.7)	122 (26.6)	
Layperson	47,409 (40.2)	269 (55.6)		1011 (55.2)	252 (55.0)	
EMS	11,961 (10.1)	87 (18.0)		296 (16.2)	84 (18.3)	
Place of arrest, public	20,118 (17.1)	193 (39.9)	0.523	686 (37.4)	180 (39.3)	0.038
*Bystander characteristics*						
Bystander CPR	64,455 (54.7)	268 (55.4)	0.014	1025 (55.9)	248 (54.1)	0.036
Bystander defibrillation	581 (0.5)	8 (1.7)	0.113	29 (1.6)	8 (1.7)	0.013
*EMS characteristics*						
Multi-tier response	58,158 (49.3)	254 (52.5)	0.063	1029 (56.2)	235 (51.3)	0.098
Response time	7.0 [5.0, 9.0]	6.0 [5.0, 9.0]	0.173	6.0 [5.0, 8.0]	6.0 [5.0, 9.0]	0.046
Scene time	13.0 [9.0, 17.0]	13.0 [9.0, 18.0]	0.011	13.0 [9.0, 18.0]	13.0 [9.0, 18.0]	0.064
Transport time	6.0 [4.0, 10.0]	7.0 [5.0, 9.0]	0.064	6.0 [4.0, 9.0]	7.0 [5.0, 9.0]	0.007
Initial cardiac rhythm			1.248			0.037
Shockable	16,057 (13.6)	292 (60.3)		1052 (57.4)	271 (59.2)	
PEA	23,270 (19.7)	100 (20.7)		394 (21.5)	96 (21.0)	
Asystole	78,580 (66.6)	92 (19.0)		386 (21.1)	91 (19.9)	
*EMS managements*						
IV-line insertion	46,917 (39.8)	254 (52.5)	0.257	1047 (57.2)	238 (52.0)	0.104
Mechanical CPR device use	15,018 (12.7)	79 (16.3)	0.102	324 (17.7)	72 (15.7)	0.053
Advanced airway management	73,537 (62.4)	337 (69.6)	0.154	1341 (73.2)	316 (69.0)	0.093
EMS defibrillation	24,199 (20.5)	328 (67.8)	1.082	1204 (65.7)	304 (66.4)	0.014
Any ROSC prior to ED arrival	4603 (3.9)	57 (11.8)	0.296	237 (12.9)	52 (11.4)	0.048

*ECPR* Extracorporeal cardiopulmonary resuscitation; *SD* Standard deviation; *SMD* Standardised mean difference; *EMS* Emergency medical service; *CPR* Cardiopulmonary resuscitation; *PEA* Pulseless electrical activity; *IV* Intravenous; *ED* Emergency department; *ROSC* Return of spontaneous circulation

The characteristics of ECPR patients in the entire cohort and matched cohort were similar in both groups. In the matched cohort, the time from the EMS call to turn on the ECMO pump took a median of 74.0 min, and 357 (77.9%) ECPRs were done in the ED. Additionally, 176 (38.4%) of ECPR patients received reperfusion therapy and 77 (16.8%) received targeted temperature management (Table 2).

**Table 2:** ECPR patient characteristics of the entire cohort and matched cohort

	Entire cohort	Matched cohort
Total ECPR	*N* = 484	*N* = 458
Time from EMS call to ECMO pump-on, median (IQR) [min]	74.0 [61.0, 92.0]	74.0 [61.0, 91.0]
Time from EMS call to ED arrival, median (IQR) [min]	28.0 [22.0, 34.0]	28.0 [22.0, 34.0]
Time from ED arrival to ECMO pump-on, median (IQR) [min]	45.5 [35.0, 62.0]	44.0 [35.0, 61.8]
Total ECLS duration, median (IQT) [days]	0.9 [0.2, 3.0]	1.0 [0.2, 3.0]
ECPR location		
ED	375 (77.5)	357 (77.9)
CAG room	101 (20.9)	93 (20.3)
Others	8 (1.7)	8 (1.7)
Other hospital care		
Reperfusion therapy	187 (38.6)	176 (38.4)
Targeted temperature management	83 (17.1)	77 (16.8)

*ECPR* Extracorporeal cardiopulmonary resuscitation; *ECMO* Extracorporeal membrane oxygenation; *EMS* Emergency medical service; *ED* Emergency department; *ECLS* Extracorporeal life support; *CAG* Coronary angiography

### Main analysis

ECPR was positively associated with good neurological recovery (ECPR: 9.9% [48/484] vs. no ECPR: 1.3% [1568/117,907]; RR 7.46 [95% CI 5.68–9.80]) and survival to discharge (ECPR: 14.3% [69/484] vs. no ECPR: 4.0% (4707/117,907); RR 3.57 [95% CI 2.87–4.45]) in the entire cohort. However, in the matched cohort, ECPR was not associated with good neurological recovery (ECPR: 10.3% [47/458] vs. no ECPR: 6.9% [127/1832]; RR 1.28 [95% CI 0.85–1.93]) or survival to discharge (ECPR: 14.6% [67/458] vs. no ECPR: 13.9% [254/1832]; RR 1.05 [95% CI 0.82–1.35]) (Table 3).

**Table 3:** Patient outcomes by ECPR in the entire cohort and matched cohort

	Entire Cohort			Matched Cohort		
	No ECPR	ECPR	RR (95% CI)	No ECPR	ECPR	RR (95% CI)
	*n*/*N* (%)	*n*/*N* (%)		*n*/*N* (%)	*n*/*N* (%)	
*Primary outcome*						
Good neurological recovery	1568/117,907 (1.3%)	48/484 (9.9%)	7.46 (5.68–9.80)	127/1832 (6.9%)	47/458 (10.3%)	1.28 (0.85–1.93)
*Secondary outcome*						
Survival to discharge	4707/117,907 (4.0%)	69/484 (14.3%)	3.57 (2.87–4.45)	254/1832 (13.9%)	67/458 (14.6%)	1.05 (0.82–1.35)

*ECPR* Extracorporeal cardiopulmonary resuscitation; *RR* Risk ratio; *CI* Confidence interval

Table 4 presents the results of the stratified analyses of the matched cohorts according to the timing of matching. Earlier ECPR within 45 min was associated with favourable neurological outcomes (ECPR: 17.3% [13/75] vs. no ECPR: 7.0% [21/300]; RR 2.51 [95% CI 1.33–4.75] in the 1–30 min group, ECPR: 13.3% [21/158] vs. no ECPR: 7.4% [47/632]; RR 1.81 [95% CI 1.11–2.93] in the 31–45 min group). A similar tendency was observed for the point estimates of survival to discharge, but no differences were statistically significant according to the stratified timing of matching.

**Table 4:** Stratified analyses by the timing of matching in matched cohort

	No ECPR	ECPR	Risk ratio (95% CI)
	*n*/*N* (%)	*n*/*N* (%)	
*Good neurological recovery*			
Timing: 01–30 min	21/300 (7.0%)	13/75 (17.3%)	2.51 (1.33–4.75)
Timing: 31–45 min	47/632 (7.4%)	21/158 (13.3%)	1.81 (1.11–2.93)
Timing: 46–60 min	41/604 (6.8%)	11/151 (7.3%)	1.07 (0.56–2.04)
Timing: 61- min	18/296 (6.1%)	2/74 (2.7%)	0.45 (0.11–1.91)
*Survival to discharge*			
Timing: 01–30 min	48/300 (16.0%)	20/75 (26.7%)	1.78 (0.50–6.43)
Timing: 31–45 min	94/632 (14.9%)	29/158 (18.4%)	1.23 (0.85–1.79)
Timing: 46–60 min	75/604 (12.4%)	13/151 (8.6%)	0.69 (0.39–1.21)
Timing: 61- min	37/296 (12.5%)	5/74 (6.8%)	0.54 (0.22–1.33)

*ECPR* Extracorporeal cardiopulmonary resuscitation; *CI* Confidence interval

In stratified analysis with initial cardiac rhythm, ECPR showed a positive effect on good neurological recovery in shockable rhythm (RR 1.70 [95% CI 1.10–2.62]). However, there was no significant difference in good neurological recovery between the ECPR and the no ECPR groups in non-shockable rhythm (RR 1.31 [95% CI 0.32–5.31]) (Table 5). Sensitivity analysis with additional adjustment for EMS arrival at the scene to ED arrival time showed a similar result (data not presented).

**Table 5:** Patient outcomes by ECPR in the matched cohort according to the initial cardiac rhythm

Survival outcomes	Initial cardiac rhythm	No ECPR	ECPR	RR (95% CI)
		*n*/*N* (%)	*n*/*N* (%)	
*Good neurological recovery*				
Shockable rhythm		64/440 (14.5%)	27/110 (24.5%)	1.70 (1.10–2.62)
Non-shockable rhythm		3/208 (1.4%)	1/52 (1.9%)	1.31 (0.32–5.31)
*Survival to discharge*				
Shockable rhythm		104/440 (23.6%)	33/110 (30.0%)	1.27 (0.91–1.77)
Non-shockable rhythm		11/208 (5.3%)	1/52 (1.9%)	0.36 (0.47–2.75)

*ECPR* Extracorporeal cardiopulmonary resuscitation; *RR* Risk ratio; *CI* Confidence interval

## Discussion

In this study, using a prospective, nationwide, population-based OHCA registry with time-dependent propensity score matching analysis, we found that ECPR was not associated with good neurological recovery at discharge; however, stratified analysis by the timing of matching showed that early ECPR (ECPR with a pump-on within 45 min after ED arrival) was positively associated with good neurological recovery in adults with OHCA of a medical cause.

We could not confirm the positive effects of ECPR. Although controversial, some previous studies have reported the effectiveness of ECPR [34, 35]. Since ECPR is performed in highly selected patients, it would be difficult to sufficiently adjust for the different characteristics between the ECPR and no ECPR groups. In addition, because of the time-sensitive nature of OHCA, resuscitation time bias can affect the results of the study. The overall effect of time-sensitive intervention for OHCA such as ECPR could be diluted when analysed over all resuscitation times. After reducing these biases through time-dependent propensity score matching analysis, we found that only early ECPR was associated with good neurological recovery. The favourable outcome of early ECMO initiation is consistent with previous findings [36, 37]. The benefits of earlier procedure initiation in time-sensitive interventions are also known for other diseases [17, 38–40]. Identifying a specific time window for which interventions are beneficial is also important.

The recent ARREST trial, a single-centre randomised controlled trial directly comparing ECPR and CPR, showed a higher survival rate in the ECPR group (43%) than in the CPR group (7%) [7]. This study applied strict inclusion and exclusion criteria for the start of ECMO (patients with a shockable rhythm, no ROSC after the defibrillation shock, estimated transfer time of less than 30 min). Another recent randomised clinical trial was conducted at a single centre in the Czech Republic and compared invasive strategies, including mechanical CPR, ECPR, and immediate invasive treatment, in the management of refractory OHCA. The study found no statistically significant difference in survival outcomes between the invasive strategy and standard strategy groups [41]. In this study, 66% of patients undergoing an invasive strategy received ECPR and 8% of those undergoing a standard strategy received ECPR. Various biases can affect the analysis of the effectiveness of ECPR across studies. Regarding the two previous randomised controlled trials, only patients with a shockable rhythm were included in the ARREST trial, as opposed to the Czech study, which was known to show a greater effectiveness of ECPR [42]. In the ARREST trial, patients were randomised after arrival at the hospital, in contrast to the Czech study, in which randomisation was performed in the prehospital stage. Including only patients who arrived at the hospital might affect the generalisability of study findings.

It is crucial to reduce the time required for ECPR in eligible patients. In the Czech study, close cooperation of the EMS dispatch centre would have contributed to the hospitals preparing ECMO earlier than usual settings [41]. The time of implantation (door to ECPR) in the intervention group was 12 (9–15) min in the study. Prehospital ECPR implementation is another promising strategy for reducing the time to ECPR. Feasibility studies showed that the ECPR time could be reduced to less than 30 min in the prehospital stage [43, 44]. Reducing the duration of ECPR can also contribute to increasing the number of ECPR targets because the beneficial effect of ECPR can be increased when applied early.

Our study included significant proportion of patients with pulseless electrical activity (21.5% in the no ECPR group and 20.2% in the ECPR group), asystole (21.1% in the no ECPR group and 19.9% in the ECPR group), and unwitnessed OHCA (28.7% in the no ECPR group and 26.6% in the ECPR group), which is less common in other studies [6, 7, 34, 35]. Although the entire cohort included 484 ECPR patients and 458 patients were included in the matched cohort, the characteristics of patients who received ECPR were similar between the entire ECPR cohort and matched ECPR cohort (Tables 1 and 2). Diverse characteristics of the study population of this study might form some basis for extending the indications for ECPR. Although we found that the benefit of ECPR was more prominent in shockable patients, recent studies have shown that ECPR might be effective even in non-shockable patients [45, 46]. Because ECPR is resource-intensive, further research is warranted to reduce the time to ECPR and explore target OHCA patient populations that can benefit [47].

### Limitations

This study had several limitations. First, because national data were used, there was no uniform ECPR protocol among hospitals and no precise information about ECPR, such as the skill level or area of practitioner specialisation. Second, the complications of ECPR were unknown due to the unavailability of the data. Third, owing to the small number of ECPR patients, a subgroup analysis according to patient characteristics could not be performed. Particularly, separate analyses according to the ECPR volume of each hospital could not be performed, which might be related to the quality of intervention. The effect of ECPR might also vary depending on the volume and the quality of EPCR in the hospital. In addition, because there were not enough patients at either end of the period, matching was not performed for each minute, and certain periods were grouped into time spans. Fifth, there could have been unmeasured bias among the groups. Sixth, because emergency medical systems are built differently according to their communities, generalising the results to other countries is constrained. In addition, because ECPR is administered only to selected patients and since the selection criteria are centre -specific, the present study results cannot be generalised to all OHCA patients. Moreover, since we performed our analysis on OHCA patients with medical causes, our findings should not be generalised to OHCA patients with other causes.

## Conclusions

The study found that ECPR was not associated with good neurological recovery at discharge; however, early ECPR was positively associated with good neurological recovery in adult OHCA patients with medical causes. A significant proportion of asystole and unwitnessed cases were included in our study, but the overall effect of early EPCR was maintained. Efforts to implement early ECPR and expansion of target group for effective ECPR are warranted.

## Data Availability

The authors participated in the data collection and quality control process as a team of researchers in the Korea OHCA registry construction project. However, ownership of the final database is held by the Korea Disease Control and Prevention Agency who have authority for the Korea OHCA registry dataset in Korea. Permission is required to use the dataset and can be requested by website (website: https://www.kdca.go.kr/injury/biz/injury/main/mainPage.do).

## Abbreviations

CI: Confidence interval
DNR: Do not resuscitate
DOA: Death on arrival
ECLS: Extracorporeal life support
ECPR: Extracorporeal cardiopulmonary resuscitation
ED: Emergency department
EMS: Emergency medical service
GEE: Generalised estimating equation
IRB: Institutional review board
OHCA: Out-of-hospital cardiac arrest
ROSC: Return of spontaneous circulation
RR: Risk ratio
VA-ECMO: Venoarterial extracorporeal membrane oxygenation

## References

[1] Kennedy JH (1966). The role of assisted circulation in cardiac resuscitation. JAMA.

[2] MacLaren G, Masoumi A, Brodie D (2020). ECPR for out-of-hospital cardiac arrest: more evidence is needed. Crit Care.

[3] Holmberg MJ, Geri G, Wiberg S, Guerguerian AM, Donnino MW, Nolan JP, Deakin CD, Andersen LW (2018). International liaison committee on resuscitation's advanced life S, pediatric task F: Extracorporeal cardiopulmonary resuscitation for cardiac arrest: a systematic review. Resuscitation.

[4] Ahn C, Kim W, Cho Y, Choi KS, Jang BH, Lim TH (2016). Efficacy of extracorporeal cardiopulmonary resuscitation compared to conventional cardiopulmonary resuscitation for adult cardiac arrest patients: a systematic review and meta-analysis. Sci Rep.

[5] Chen Z, Liu C, Huang J, Zeng P, Lin J, Zhu R, Lu J, Zhou Z, Zuo L, Liu G (2019). Clinical efficacy of extracorporeal cardiopulmonary resuscitation for adults with cardiac arrest: meta-analysis with trial sequential analysis. Biomed Res Int.

[6] Belohlavek J, Smalcova J, Rob D, Franek O, Smid O, Pokorna M, Horak J, Mrazek V, Kovarnik T, Zemanek D (2022). Effect of intra-arrest transport, extracorporeal cardiopulmonary resuscitation, and immediate invasive assessment and treatment on functional neurologic outcome in refractory out-of-hospital cardiac arrest: a randomized clinical trial. JAMA.

[7] Yannopoulos D, Bartos J, Raveendran G, Walser E, Connett J, Murray TA, Collins G, Zhang L, Kalra R, Kosmopoulos M (2020). Advanced reperfusion strategies for patients with out-of-hospital cardiac arrest and refractory ventricular fibrillation (ARREST): a phase 2, single centre, open-label, randomised controlled trial. Lancet.

[8] Miraglia D, Miguel LA, Alonso W (2020). Extracorporeal cardiopulmonary resuscitation for in- and out-of-hospital cardiac arrest: systematic review and meta-analysis of propensity score-matched cohort studies. J Am Coll Emerg Phys Open.

[9] Austin PC (2011). An introduction to propensity score methods for reducing the effects of confounding in observational studies. Multivar Behav Res.

[10] Patricio D, Peluso L, Brasseur A, Lheureux O, Belliato M, Vincent JL, Creteur J, Taccone FS (2019). Comparison of extracorporeal and conventional cardiopulmonary resuscitation: a retrospective propensity score matched study. Crit Care.

[11] Andersen LW, Grossestreuer AV, Donnino MW (2018). "Resuscitation time bias"-a unique challenge for observational cardiac arrest research. Resuscitation.

[12] Reynolds JC, Grunau BE, Rittenberger JC, Sawyer KN, Kurz MC, Callaway CW (2016). Association between duration of resuscitation and favorable outcome after out-of-hospital cardiac arrest: implications for prolonging or terminating resuscitation. Circulation.

[13] Lu B (2005). Propensity score matching with time-dependent covariates. Biometrics.

[14] Schisterman EF, Cole SR, Platt RW (2009). Overadjustment bias and unnecessary adjustment in epidemiologic studies. Epidemiology.

[15] Andersen LW, Raymond TT, Berg RA, Nadkarni VM, Grossestreuer AV, Kurth T, Donnino MW (2016). Association between tracheal intubation during pediatric in-hospital cardiac arrest and survival. JAMA.

[16] Zhang Z, Li X, Wu X, Qiu H, Shi H (2020). written on behalf of AMEB-DCTCG: propensity score analysis for time-dependent exposure. Ann Transl Med.

[17] Wengenmayer T, Rombach S, Ramshorn F, Biever P, Bode C, Duerschmied D, Staudacher DL (2017). Influence of low-flow time on survival after extracorporeal cardiopulmonary resuscitation (eCPR). Crit Care.

[18] Oh J, Cha KC, Lee JH, Park S, Kim DH, Lee BK, Park JS, Jung WJ, Lee DK, Roh YI (2021). 2020 Korean guidelines for cardiopulmonary resuscitation. Part 4. Adult advanced life support. Clin Exp Emerg Med.

[19] Panchal AR, Bartos JA, Cabanas JG, Donnino MW, Drennan IR, Hirsch KG, Kudenchuk PJ, Kurz MC, Lavonas EJ, Morley PT (2020). Part 3: adult basic and advanced life support: 2020 American heart association guidelines for cardiopulmonary resuscitation and emergency cardiovascular care. Circulation.

[20] Ha TS, Yang JH, Cho YH, Chung CR, Park CM, Jeon K, Suh GY (2017). Clinical outcomes after rescue extracorporeal cardiopulmonary resuscitation for out-of-hospital cardiac arrest. Emerg Med J.

[21] Cho YS, Song KH, Lee BK, Jeung KW, Jung YH, Lee DH, Lee SM (2017). Five-year experience of extracorporeal life support in emergency physicians. Korean J Crit Care Med.

[22] Ryu JA, Cho YH, Sung K, Choi SH, Yang JH, Choi JH, Lee DS, Yang JH (2015). Predictors of neurological outcomes after successful extracorporeal cardiopulmonary resuscitation. BMC Anesthesiol.

[23] Ro YS, Shin SD, Song KJ, Lee EJ, Kim JY, Ahn KO, Chung SP, Kim YT, Hong SO, Choi J-A et al. A trend in epidemiology and outcomes of out-of-hospital cardiac arrest by urbanization level: a nationwide observational study from 2006 to 2010 in South Korea. In: Resuscitation*.* vol. 84. Elsevier; 2013. pp. 547–557. 10.1016/j.resuscitation.2012.12.020 23313428

[24] Kim YT, Shin SD, Hong SO, Ahn KO, Ro YS, Song KJ, Hong KJ. Effect of national implementation of utstein recommendation from the global resuscitation alliance on ten steps to improve outcomes from Out-of-Hospital cardiac arrest: a ten-year observational study in Korea. In: BMJ Open*.* vol. 7. British Medical Journal Publishing Group; 2017. 10.1136/bmjopen-2017-016925 PMC5724141 28827263

[25] Becker LB, Aufderheide TP, Geocadin RG, Callaway CW, Lazar RM, Donnino MW, Nadkarni VM, Abella BS, Adrie C, Berg RA (2011). Primary outcomes for resuscitation science studies: a consensus statement from the American Heart Association. Circulation.

[26] Raina KD, Callaway C, Rittenberger JC, Holm MB (2008). Neurological and functional status following cardiac arrest: method and tool utility. Resuscitation.

[27] Andersen LW, Granfeldt A, Callaway CW, Bradley SM, Soar J, Nolan JP, Kurth T, Donnino MW (2017). American Heart Association's get with the guidelines-resuscitation I: association between tracheal intubation during adult in-hospital cardiac arrest and survival. JAMA.

[28] Okubo M, Komukai S, Izawa J, Gibo K, Kiyohara K, Matsuyama T, Kiguchi T, Iwami T, Callaway CW, Kitamura T (2019). Prehospital advanced airway management for paediatric patients with out-of-hospital cardiac arrest: a nationwide cohort study. Resuscitation.

[29] Hansen BB, Klopfer SO (2006). Optimal full matching and related designs via network flows. J Comput Graph Stat.

[30] Normand ST, Landrum MB, Guadagnoli E, Ayanian JZ, Ryan TJ, Cleary PD, McNeil BJ (2001). Validating recommendations for coronary angiography following acute myocardial infarction in the elderly: a matched analysis using propensity scores. J Clin Epidemiol.

[31] Stuart EA, Lee BK, Leacy FP (2013). Prognostic score-based balance measures can be a useful diagnostic for propensity score methods in comparative effectiveness research. J Clin Epidemiol.

[32] Zeger SL, Liang KY (1986). Longitudinal data analysis for discrete and continuous outcomes. Biometrics.

[33] Park JH, Song KJ, Shin SD, Ro YS, Hong KJ (2019). Time from arrest to extracorporeal cardiopulmonary resuscitation and survival after out-of-hospital cardiac arrest. Emerg Med Australas.

[34] Alfalasi R, Downing J, Cardona S, Lowie BJ, Fairchild M, Chan C, Powell E, Pourmand A, Grazioli A, Tran QK (2022). A comparison between conventional and extracorporeal cardiopulmonary resuscitation in out-of-hospital cardiac arrest: a systematic review and meta-analysis. Healthcare (Basel).

[35] Downing J, Al Falasi R, Cardona S, Fairchild M, Lowie B, Chan C, Powell E, Pourmand A, Tran QK (2022). How effective is extracorporeal cardiopulmonary resuscitation (ECPR) for out-of-hospital cardiac arrest? A systematic review and meta-analysis. Am J Emerg Med.

[36] Debaty G, Babaz V, Durand M, Gaide-Chevronnay L, Fournel E, Blancher M, Bouvaist H, Chavanon O, Maignan M, Bouzat P (2017). Prognostic factors for extracorporeal cardiopulmonary resuscitation recipients following out-of-hospital refractory cardiac arrest. A systematic review and meta-analysis. Resuscitation.

[37] Fagnoul D, Combes A, De Backer D (2014). Extracorporeal cardiopulmonary resuscitation. Curr Opin Crit Care.

[38] Marini JJ (2017). Time-sensitive therapeutics. Crit Care.

[39] Seymour CW, Gesten F, Prescott HC, Friedrich ME, Iwashyna TJ, Phillips GS, Lemeshow S, Osborn T, Terry KM, Levy MM (2017). Time to treatment and mortality during mandated emergency care for sepsis. N Engl J Med.

[40] Papazian L, Forel J-M, Gacouin A, Penot-Ragon C, Perrin G, Loundou A, Jaber S, Arnal J-M, Perez D, Seghboyan J-M (2010). Neuromuscular blockers in early acute respiratory distress syndrome. N Engl J Med.

[41] Belohlavek J, Smalcova J, Rob D, Franek O, Smid O, Pokorna M, Horák J, Mrazek V, Kovarnik T, Zemanek D (2022). Effect of intra-arrest transport, extracorporeal cardiopulmonary resuscitation, and immediate invasive assessment and treatment on functional neurologic outcome in refractory out-of-hospital cardiac arrest: a randomized clinical trial. JAMA.

[42] Bartos JA, Grunau B, Carlson C, Duval S, Ripeckyj A, Kalra R, Raveendran G, John R, Conterato M, Frascone RJ (2020). Improved survival with extracorporeal cardiopulmonary resuscitation despite progressive metabolic derangement associated with prolonged resuscitation. Circulation.

[43] Singer B, Reynolds JC, Davies GE, Wrigley F, Whitbread M, Faulkner M, O’Brien B, Proudfoot AG, Mathur A, Evens T (2020). Sub30: protocol for the Sub30 feasibility study of a pre-hospital Extracorporeal membrane oxygenation (ECMO) capable advanced resuscitation team at achieving blood flow within 30 min in patients with refractory out-of-hospital cardiac arrest. Resusc Plus.

[44] Singer B, Reynolds JC, Davies GE, Wrigley F, Whitbread M, Faulkner M, O'Brien B, Proudfoot AG, Mathur A, Evens T (2020). Sub30: Protocol for the Sub30 feasibility study of a pre-hospital Extracorporeal membrane oxygenation (ECMO) capable advanced resuscitation team at achieving blood flow within 30 min in patients with refractory out-of-hospital cardiac arrest. Resusc Plus.

[45] Shirasaki K, Hifumi T, Goto M, Shin K, Horie K, Isokawa S, Inoue A, Sakamoto T, Kuroda Y, Imai R (2023). Clinical characteristics and outcomes after extracorporeal cardiopulmonary resuscitation in out-of-hospital cardiac arrest patients with an initial asystole rhythm. Resuscitation.

[46] Tanimoto A, Sugiyama K, Tanabe M, Kitagawa K, Kawakami A, Hamabe Y (2020). Out-of-hospital cardiac arrest patients with an initial non-shockable rhythm could be candidates for extracorporeal cardiopulmonary resuscitation: a retrospective study. Scand J Trauma Resusc Emerg Med.

[47] Karve S, Lahood D, Diehl A, Burrell A, Tian DH, Southwood T, Forrest P, Dennis M (2021). The impact of selection criteria and study design on reported survival outcomes in extracorporeal oxygenation cardiopulmonary resuscitation (ECPR): a systematic review and meta-analysis. Scand J Trauma Resusc Emerg Med.

